# Multi-Target Tracking with Collaborative Roadside Units Under Foggy Conditions

**DOI:** 10.3390/s26030998

**Published:** 2026-02-03

**Authors:** Tao Shi, Xuan Wang, Wei Jiang, Xiansheng Huang, Ming Cen, Shuai Cao, Hao Zhou

**Affiliations:** 1 State Key Laboratory of Intelligent Vehicle Safety Technology, Chongqing 400023, China; st_swjtu@163.com; 2 Intelligent Connected Vehicle Inspection Center (Hunan) of CAERI Co., Ltd., Changsha 410205, China; wangxuan@caeri.com.cn (X.W.); huangxiansheng@caeri.com.cn (X.H.); 99992189@caeri.com.cn (S.C.); 99993965@caeri.com.cn (H.Z.); 3 Jiangsu CAERI Automotive Engineering Research Institute Co., Ltd., Suzhou 215151, China; 4 School of Automation, Chongqing University of Posts and Telecommunications, Chongqing 400065, China; m_cen0104@sina.com

**Keywords:** collaborative RSU, roadside LiDAR, foggy conditions, LiDAR denoising, multi-target tracking, particle PHD filter

## Abstract

The Intelligent Road Side Unit (RSU) is a crucial component of Intelligent Transportation Systems (ITSs), where roadside LiDAR are widely utilized for their high precision and resolution. However, water droplets and atmospheric particles in fog significantly attenuate and scatter LiDAR beams, posing a challenge to multi-target tracking and ITS safety. To enhance the accuracy and reliability of RSU-based tracking, a collaborative RSU method that integrates denoising and tracking for multi-target tracking is proposed. The proposed approach first dynamically adjusts the filtering kernel scale based on local noise levels to effectively remove noisy point clouds using a modified bilateral filter. Subsequently, a multi-RSU cooperative tracking framework is designed, which employs a particle Probability Hypothesis Density (PHD) filter to estimate target states via measurement fusion. A multi-target tracking system for intelligent RSUs in Foggy scenarios was designed and implemented. Extensive experiments were conducted using an intelligent roadside platform in real-world fog-affected traffic environments to validate the accuracy and real-time performance of the proposed algorithm. Experimental results demonstrate that the proposed method improves the target detection accuracy by 8% and 29%, respectively, compared to statistical filtering methods after removing fog noise under thin and thick fog conditions. At the same time, this method performs well in tracking multi-class targets, surpassing existing state-of-the-art methods, especially in high-order evaluation indicators such as HOTA, MOTA, and IDs.

## 1. Introduction

In recent years, breakthrough advances in artificial intelligence have accelerated the development of ITS [1], facilitating their transition from theoretical research to practical implementation. However, single-vehicle perception systems are often inadequate in complex traffic environments due to limitations in the field of view of onboard sensors and constrained computational resources. To address these challenges, Vehicle-Infrastructure Cooperative Systems (VICS) [2] have been introduced, which leverage roadside units (RSUs) [3] and vehicle-mounted terminals for collaborative environmental sensing. Such systems provide autonomous vehicles with beyond-line-of-sight environmental information, thereby substantially enhancing safety redundancy and scene adaptability.

Within VICS, RSUs employ a variety of sensors for environmental perception. Although visual sensors (e.g., cameras) offer advantages in terms of low cost and high resolution, their imaging quality is susceptible to abrupt illumination changes and adverse weather conditions. Although millimeter-wave radar is capable of all-weather operation and exhibits strong anti-interference characteristics, it is limited by its relatively low angular resolution, making it difficult to accurately discern the geometric features of targets. In contrast, LiDAR (Light Detection and Ranging), with its wide-area detection capability and centimeter-level ranging accuracy, enables the construction of high-resolution 3D environmental point clouds and has thus become a core sensor in RSU-based environmental perception.

Fog is a frequently encountered meteorological condition, particularly in mountainous regions. Taking ChengDu as an example, fog occurs on approximately 125 days annually, posing significant challenges to target detection and tracking by RSUs. For instance, cameras suffer from reduced image contrast and a marked increase in noise due to light scattering in fog [4]. Millimeter-wave radar is affected by water vapor absorption peaks near harmonics such as 60 GHz, leading to considerable attenuation in signal strength and angular resolution [5,6]. As for LiDAR, suspended particles in fog—such as water droplets and aerosols—induce Mie scattering of laser beams, resulting in path loss and directional deviation of the transmitted signals [7,8]. This physical phenomenon leads to spatially heterogeneous degradation of point cloud quality: at long ranges, scattering significantly reduces point density, causing target contours to become blurred or even undetectable; at close ranges, multiple scattering generates numerous “ghost” noise points. These spurious points interweave with legitimate returns, substantially complicating target detection and motion state estimation, thereby imposing more stringent requirements on the robustness of multi-object tracking algorithms.

Subsequently, we critically review classical LiDAR point cloud denoising methods (e.g., statistical outlier removal and radius-based filtering), highlighting their fundamental limitation in handling dynamic noise under high-clutter scenarios. Thereafter, we introduce Multi-Object Tracking (MOT) technology through two distinct paradigms—data association frameworks (e.g., Kalman filter-based tracking) and Random Finite Set (RFS) theory. This theoretical foundation directly enables our Particle PHD filter design in Chapter 3, which resolves trajectory fragmentation in real-time denoising.

### 1.1. Lidar Point Cloud Denoising Method in Foggy Conditions

The adverse effects of fog on LiDAR systems primarily manifest as signal attenuation and point cloud noise [9]. Dense fog particles cause significant laser signal attenuation due to Mie scattering, with severity increasing at higher fog densities and shorter wavelengths. Backscattering from fog particles introduces false returns that corrupt point cloud data, while multiple scattering extends optical paths, distorts waveforms, and increases ranging errors. These phenomena collectively degrade point cloud quality and measurement accuracy, posing fundamental challenges to reliable LiDAR perception in adverse weather conditions. For this reason, point cloud denoising methods must be used to dynamically suppress the fog noise point cloud and guarantee the robustness of the downstream sensing algorithm. Currently commonly used point cloud denoising methods are as follows.

Point cloud denoising in perception systems primarily relies on three categories of methods: statistical filtering, deep learning, and multi-sensor fusion. Statistical filtering techniques, such as Gaussian [10], mean [11], and median [12,13] filtering, smooth data by leveraging statistical properties within local neighborhoods. They are computationally simple and efficient, yet exhibit limited adaptability to complex noise patterns. Deep learning approaches, including CNN [14] and GNN [15]-based models like PointNet [16] and PointNet++ [17], automatically learn both local and global features from point clouds, effectively handling diverse noise types and demonstrating strong generalization capabilities. However, they often require large annotated datasets and entail higher computational costs. Multi-sensor fusion methods improve robustness and accuracy under low-visibility conditions by integrating complementary information from heterogeneous sensors such as LiDAR, cameras, and millimeter-wave radar, often supported by filtering techniques like Kalman [18] or particle [19] filtering. These strategies collectively highlight a trend toward leveraging complementary information to overcome the limitations of single-modality sensing.

### 1.2. Multi-Target Tracking Methods

MOT [20] serves as a critical technology in ITS, designed to achieve continuous localization, identity maintenance, and trajectory estimation of multiple targets in dynamic environments using sequential sensor data. Existing research methodologies can be broadly categorized into two frameworks: data association and Random Finite Set (RFS)-based approaches [21].

#### 1.2.1. Data Association

MOT based on data association establishes correspondences between sensor measurements and target states, facing challenges such as occlusion, clutter, and dynamic scenarios. Classical approaches are divided into probabilistic and optimal assignment categories. Probabilistic methods include Nearest Neighbor (NN) [22], Probabilistic Data Association (PDA) [23], and JPDA [24], with the latter suffering from combinatorial complexity. Multi-Hypothesis Tracking (MHT) [25] maintains multiple trajectory hypotheses but demands substantial computation. Optimal assignment methods, primarily using the Hungarian algorithm, achieve global matching, with recent improvements addressing occlusion through Kalman prediction and scene partitioning. Deep learning has promoted end-to-end frameworks like FairMOT [20] and TransTrack [26,27], which integrate detection and Re-ID tasks, reducing identity switches. However, data association methods remain constrained by their dependency on detection accuracy, limited nonlinear motion handling, and scalability issues in multi-RSU edge deployments.

#### 1.2.2. Random Finite Set

In contrast to traditional multi-target tracking methods that rely on data association and fixed target numbers—often leading to errors in roadside LiDAR monitoring due to occlusion, noise, and dynamic changes—Random Finite Set (RFS)-based approaches model all target states as a set, enabling joint estimation of target states and cardinality without pre-defined target numbers. This allows adaptive handling of target appearance, disappearance, and partial occlusion.

The RFS framework, pioneered by Mahler, recursively updates the multi-target state within a Bayesian formulation, treating target states as a whole rather than associating measurements individually. The (PHD) [28] filter propagates the intensity function of target states, avoiding explicit data association and offering robustness under occlusion and noisy LiDAR observations. Its extension, the Cardinalized PHD (CPHD) [29,30], jointly estimates target states and their number distribution, improving cardinality accuracy at increased computational cost. To address this, methods such as linear-complexity multi-sensor CPHD, Gaussian mixture [31,32] implementations, and gamma cardinality modeling have been proposed to enhance efficiency and adaptability.

For nonlinear and non-Gaussian scenarios, particle PHD filters approximate state distributions via weighted particles, while recent studies integrate deep reinforcement learning within a POMDP [33] framework, showing significant gains in tracking accuracy (23.6–41.8% OSPA improvement). These learning-augmented methods demonstrate potential in handling high-dimensional point cloud data and complex motion patterns.

Overall, existing point cloud denoising methods predominantly rely on the static scene assumption, which proves inadequate for modeling the spatiotemporal correlations of dynamic noise, such as dust raised by moving vehicles or the trajectories of raindrops. This shortcoming often leads to suboptimal filter threshold settings. Furthermore, current roadside unit (RSU)-based tracking solutions primarily depend on local sensor measurements. However, in Foggy conditions, the Mie scattering effect from airborne particulates introduces substantial noise into the point clouds. This phenomenon compromises the accuracy of local measurements and consequently inflates the observation error variance in tracking filters, thereby jeopardizing the safety and stability of the vehicle-infrastructure cooperative systems.

Therefore, in response to the challenge of multi-object tracking degradation in roadside LiDAR systems under Foggy conditions owing to fog-induced noise, this study proposes a collaborative multi-object tracking method for RSUs tailored to fog-affected environments. The proposed approach aims to enhance tracking accuracy and reliability under such adverse conditions.

## 2. Main Methods

To address the challenges of LiDAR point cloud noise and multi-target tracking instability in Foggy conditions, this paper proposes an integrated approach combining adaptive point cloud denoising with multi-RSU collaborative tracking.

### 2.1. System Framework: Multi-Target Tracking of Roadside Unit Coordination

**Figure 1:**
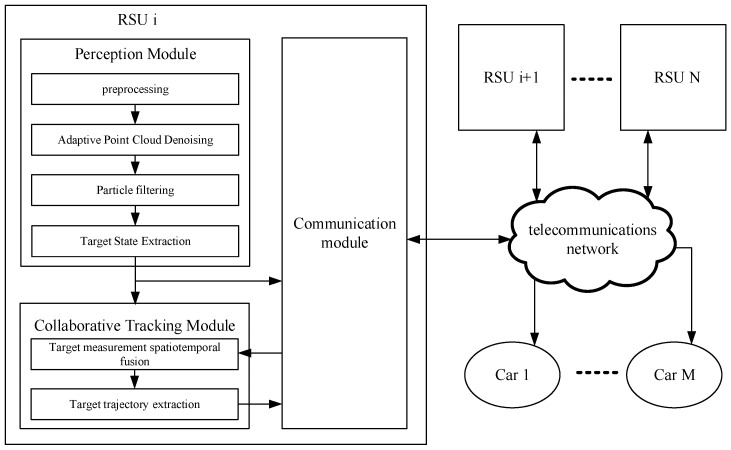
Illustration of the proposed System framework.

The proposed system framework is illustrated in Figure 1.

The system comprises a target detection module, fusion tracking module, and communication module. The target detection module preprocesses raw point clouds through background filtering and ground segmentation to derive non-ground points with residual noise, applies spatiotemporal denoising to eliminate fog-induced artifacts, and extracts targets via clustering; the fusion tracking module performs spatiotemporal fusion of road target measurements with measurement sets received from neighboring RSUs via the communication module to produce an augmented measurement set, which is processed by PHD filter to update target trajectories; the communication module enables inter-RSU measurement set exchange and delivers road traffic target information to vehicles.

### 2.2. Adaptive Lidar Point Cloud Denoising Method

**Figure 2:**
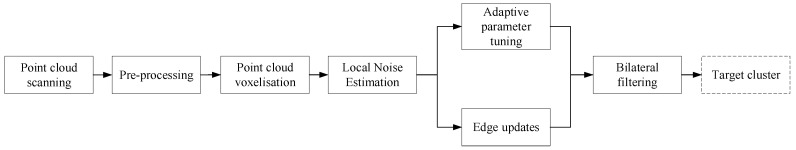
Adaptive Lidar point cloud denoising method framework.

Raw point clouds acquired from roadside LiDAR sensors are typically dense, unstructured, and contain a significant number of outliers and irrelevant points due to atmospheric interference, sensor noise, and reflection artifacts—especially in Foggy conditions. In response to the influence of fog on point clouds mentioned above, this paper proposes an adaptive LIDAR point cloud denoising method. The framework of the method is shown in Figure 2.

#### 2.2.1. Point Cloud Preprocessing

The point cloud data used in this study was scanned using RS RubyLite (v. 23071401), and each point cloud corresponds to the actual position of the physical laser reflection on the surface of the object. The RS-RubyLite LiDAR, is an 80-channel mechanical spinning LiDAR specifically engineered for medium-to-high-speed autonomous driving applications. This sensor achieved a vertical angular resolution of 0.1° and delivers a detection range of 160 m against targets with 10% reflectivity, thereby providing sufficient environmental perception capabilities for diverse operational scenarios including autonomous passenger vehicles, heavy-duty mining trucks, commercial haulage vehicles, and vehicle-infrastructure cooperative systems.

The detailed technical specifications are shown in Table 1:

Point cloud preprocessing includes region of interest segmentation, point cloud segmentation, and point cloud integration. First, interest segmentation is explained from a practical perspective as background filtering—the process of removing useless point cloud points belonging to background elements (such as road surfaces, buildings, and atmospheric particles) to isolate points from relevant targets. Pass-through filtering is applied to define a 3D region of interest, effectively narrowing the processing scope while preserving critical spatial data. Subsequently, radius filtering is implemented to eliminate isolated noise points, thereby improving point cloud reliability. Building on this, a ground segmentation method integrating grid-based height difference analysis and local plane fitting is proposed: by constructing a grid map to compute height characteristics within each unit and combining threshold-based determination, preliminary separation between ground and non-ground points is achieved. Low-height seed points are then selected from the retained areas, and iterative plane fitting is performed using Principal Component Analysis (PCA) [34] to accomplish accurate ground segmentation in complex terrain.

**Figure 3:**
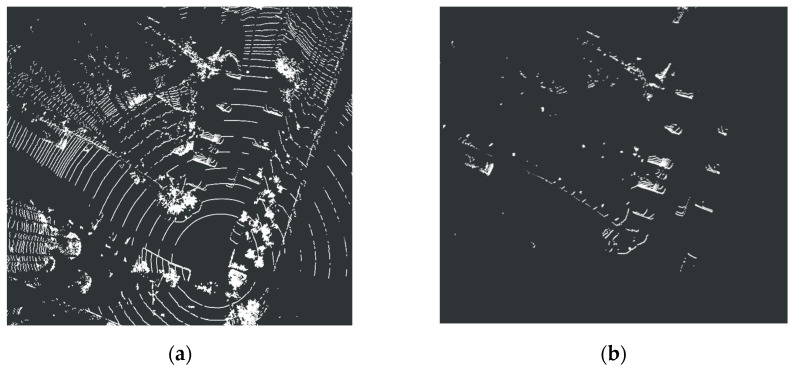
(**a**) Original point cloud. (**b**) Point cloud after preprocessing.

Point cloud density is crucial for object detection quality, as sparse point clouds will result in insufficient geometric detail capture and degraded detection performance for small or distant objects. Therefore, a method known as multi-frame point cloud fusion was further introduced, which enhanced the point density of real targets through the fusion of consecutive temporal scans while effectively suppressing random noise. Collectively, these preprocessing steps established a high-quality data foundation for subsequent target detection and tracking tasks. The result after preprocessing was shown in Figure 3.

#### 2.2.2. Voxelisation and Local Noise Estimation

**Figure 4:**
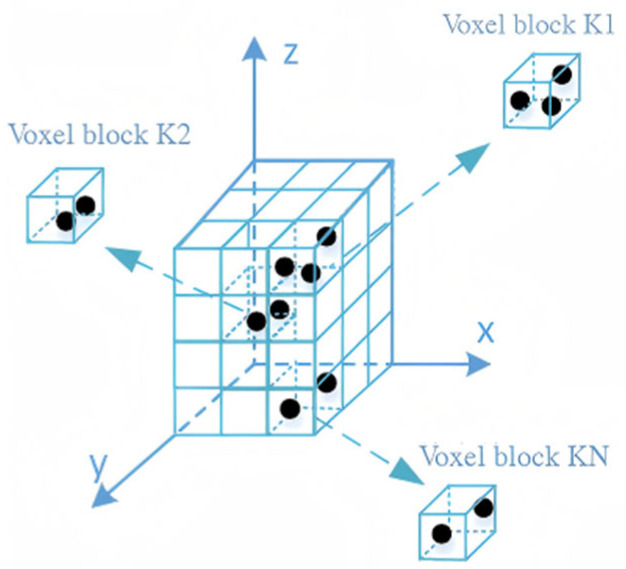
Point cloud voxelization.

Point cloud data, comprising irregularly distributed 3D points with spatial coordinates and intensity attributes, presents challenges in computational complexity and storage. Voxelization addresses this by discretizing the 3D space into uniform volumetric grids (voxels) of size $d_{x}\times d_{y}\times d_{z}$, effectively converting irregular point clouds into a structured representation. The voxelization process is shown in Figure 4. Within the roadside LiDAR coordinate system, each point p  is mapped to a specific voxel using index coordinates calculated as Equation (1).

(1) $$i=\frac{x}{d_{x}},j=\frac{y}{d_{y}},k=\frac{z}{d_{z}}$$

Leveraging the voxel structure, local noise estimation is performed efficiently. For each point $p_{i}$ within voxel V, its neighborhood $N\left(p_{i}\right)$ is defined as all points within a fixed radius r, accelerated by the voxel grid for rapid neighbor retrieval. The neighborhood point set $N\left(p_{i}\right)$ can be calculated by Equation (2):

(2) $$N\left(p_{i}\right)=\left\{p\in V|\left\|p-p_{i}\right\|\leq r\right\}$$

The local noise level is quantitatively evaluated through the spatial distribution and intensity characteristics of the neighborhood. The centroid $\mu_{v}$ and spatial standard deviation $\sigma_{v}$ are computed as

(3) $$\sigma_{v}=\sqrt{\frac{1}{N}\sum_{i=1}^{N}{\left\|p_{i}-\mu_{v}\right\|}^{2}}$$

(4) $$\mu_{v}=\frac{1}{N}\sum_{i=1}^{N}p_{i}$$

respectively. Simultaneously, the intensity mean $\mu_{I}$ and standard deviation $\sigma_{I}$ are derived as

(5) $$\sigma_{I}=\sqrt{\frac{1}{\left|N_{r}\left(p_{i}\right)\right|}\sum_{k\in N\left(p_{i}\right)}{\left(I_{k}-\mu_{I}\right)}^{2}}$$

(6) $$\mu_{I}=\frac{1}{\left|N_{r}\left(p_{i}\right)\right|}\sum_{k\in N\left(p_{i}\right)}I_{k}$$

#### 2.2.3. Parameter Adaptive Adjustment

In Foggy conditions, LiDAR point clouds exhibit a non-linear surge in noise density within close-range regions. Specifically, the heightened probability of laser scattering by suspended particles leads to an approximately exponential increase in noise points as distance decreases. Meanwhile, distant regions suffer from signal attenuation that causes noise to interlace with valid points. This section proposes a piecewise exponential mapping function defined as Equation (7):

(7) $$\sigma_{f}=\{\begin{array}{c} \alpha_{1}\cdot exp\left(\lambda \sigma_{v}\right)+\beta_{1}\;\;\;\;\sigma_{v}<\Upsilon \;\\ \alpha_{2}\cdot \sigma_{v}+\beta_{2}\;\;\;\;\;\;\;\;\;\;\;\;\;\;\;\;\;\sigma_{v}\geq \Upsilon \end{array}$$

**Remark** **1.** *In contrast to a linear mapping function, the proposed piecewise function explicitly accounts for the distinct representational characteristics of point clouds at varying distances, thereby constructing a more accurate and adaptive filtering kernel.*

The parameters α, β, γ, and τ in Equations (7) and (10) were determined through a two-step methodology: initial values were selected based on empirical experience from similar cooperative control applications reported in the literature, ensuring fundamental stability requirements were met. Subsequently, systematic fine-tuning was performed via experimental validation across multiple scenarios, where a grid search over physically meaningful ranges identified the final parameter set that optimally balanced tracking accuracy, string stability, and computational efficiency.

In low-noise regions $\left(\sigma_{v}<\Upsilon \right)$, where noise primarily manifests as isolated outliers, the exponential term $exp\left(\lambda \sigma_{v}\right)$ (with λ < 0) restrains excessive kernel expansion to preserve fine details. Parameters $\alpha_{1}$ and $\beta_{1}$ are introduced to constrain the lower bound of kernel width, ensuring fundamental denoising capability. In high-noise regions $\sigma_{v}\geq \Upsilon$, where noise becomes deeply coupled with valid points, the linear term $\alpha_{2}\cdot \sigma_{v}$ rapidly increases kernel width through slope $\alpha_{2}$ to enhance smoothing, while intercept $\beta_{2}$ compensates for background noise interference.

#### 2.2.4. Edge Updates

In 3D point cloud data structures, edge points typically exhibit significantly larger local gradient magnitudes. To preserve edge details during filtering and prevent excessive smoothing, an edge-aware updating scheme based on gradient computation is introduced. The local gradient $\nabla p_{i}$ at point $p_{i}$ is calculated as follows:

In 3D point cloud data structures, edge points are characterized by significantly larger local gradient magnitudes. To preserve these critical features during filtering and prevent undesired smoothing, an edge-aware updating scheme based on local gradient computation is introduced.

Robustness Enhancement via Pre-Smoothing: The gradient calculation in Equation (8), while computationally efficient, can be sensitive to high-frequency noise—common in foggy or low-visibility environments—as noisy points introduce random directional variations. To enhance robustness, a fast neighborhood averaging pre-processing step is applied prior to gradient computation. For each point $p_{i}$, its position is temporarily updated as the centroid of its local neighborhood $N(p_{i})$:

(8) $$\tilde{p_{i}}=\frac{1}{N(p_{i})}\sum_{k\in N\left(p_{i}\right)}p_{k}$$

The local gradient $\nabla p_{i}$ is then computed using the smoothed point $\tilde{p_{i}}$ and its smoothed neighborhood:

(9) $$\nabla p_{i}=\sum_{k\in N\left(p\right)}\frac{p_{i}-p_{k}}{\left\|p_{i}-p_{k}\right\|}$$

The gradient magnitude $\left\|\nabla p_{i}\right\|$ is then computed and compared with a predefined threshold τ to determine the edge weight:

(10) $$\omega_{i}=\{\begin{array}{c} 1+\gamma \cdot \left(\left\|\nabla p_{i}\right\|-\tau \right),\;\left\|\nabla p_{i}\right\|>\tau \\ 1,\;\;\;\;\;\;\;\;\;\;\;\;\;\;\;\;\;\;\;\;\;\;\;\;\;\;\;\;\;\;\;\;\;\left\|\nabla p_{i}\right\|<\tau \end{array}$$

**Remark** **2.** *This equation enables a soft, gradient-aware transition between smoothing and preservation, which directly enhances the denoising performance in two critical aspects: it significantly improves the retention of sharp geometric features and edges, while simultaneously preventing the over-smoothing.*

#### 2.2.5. Bilateral Filtering

In complex Foggy conditions, conventional convolution kernels struggle to simultaneously achieve noise suppression and detail preservation. To address this challenge, a bilateral filter [35] is employed for point cloud denoising, effectively balancing smoothing performance with edge protection during the filtering process. This approach enhances traditional weighted-average filtering by incorporating both spatial distance and intensity characteristics, thereby preventing the loss of edge details while removing noise.

For each target point $p_{i}$ (with spatial coordinates $p_{i}=\left(x_{i},y_{i},z_{i}\right)$ and intensity $I_{i}$) and its neighborhood set $N_{r}\left(p_{i}\right)$, the filter assigns a composite weight $\omega_{ik}$ to each neighboring point $p_{k}$. This weight integrates spatial domain, intensity domain, formulated as:

(11) $$\omega_{ik}=\omega_{i}\cdot exp\left(-\frac{{\left\|p_{i}-p_{k}\right\|}^{2}}{2\sigma_{f}^{2}}-\frac{{\left(I_{i}-I_{k}\right)}^{2}}{2\sigma_{I}^{2}}\right)$$

where $\left\|p_{i}-p_{k}\right\|$ denotes the Euclidean distance between the target point $p_{i}$ and its neighbor $p_{k}$.

After computing the bilateral filtering weights, the spatial coordinates of the target point $p_{i}$ are updated through weighted averaging to obtain the denoised coordinates. The updating formula is given by:

(12) $$p_{i}^{\prime }=\frac{\sum_{k\in N\left(p_{i}\right)}\omega_{ik}P_{k}}{\sum_{k\in N\left(p_{i}\right)}\omega_{ik}}$$

### 2.3. Multi-Object Tracking Method with Roadside Unit Collaboration

The challenges of cross-domain multi-target tracking in LiDAR systems are primarily attributed to adverse weather conditions and long-range sensing. While Section 3.1 mitigated the former via an adaptive point cloud denoising approach that effectively suppresses noise by fusing local noise statistics and intensity information, the latter persists as a critical limitation. Specifically, LiDAR’s angular resolution degrades geometrically with increasing range, yielding excessively sparse point clouds for distant targets that impede precise geometric reconstruction and stable motion characterization. Moreover, the signal-to-noise ratio deteriorates significantly at extended ranges. To overcome these limitations, we propose a cooperative multi-target tracking framework leveraging multiple RSUs.

Each RSU integrates three functional modules: perception, cooperative tracking, and communication. Within this cross-domain cooperative architecture, RSUs exchange local measurement data with adjacent units. The received measurements are subsequently fused with local observations to generate a unified observation set. A Particle PHD filter is then deployed locally at each RSU to execute multi-target tracking. Each particle is assigned a unique identifier (UID), enabling discrete target identity management. A predicted particle set is generated based on a predefined state transition model to estimate prospective target states. Particle weights are subsequently updated via the observation likelihood function, thereby ensuring accurate representation of the correspondence between particles and actual measurements.

#### 2.3.1. Measurement Fusion

In the proposed tracking framework, target measurements are provided by roadside LiDAR units. Thus, the coordinate system of the LiDAR group is adopted as the reference, and conventional spatial synchronization methods are employed to perform spatial registration among multiple LiDAR devices, thereby ensuring the continuity of target measurement coordinates.

To facilitate subsequent particle filtering, the point cloud distribution is represented using a particle model during measurement fusion (i.e., particle measurement), thereby minimizing additional computational overhead in the tracking process.

(13) $$P={\left\{\left(x_{i},\omega_{i}\right)\right\}}_{i=1}^{N},\;x_{i}\in R^{3},\;\omega_{i}\in \left[0,\;1\right]$$

where $x_{i}$ denotes the particle position and $\omega_{i}$ denotes the weight.

Let the measurement particle set from a neighboring RSU be denoted as $P_{A}=\left\{\left(x_{A_{i}},\omega_{A_{i}}\right)\right\}$, and the local particle set as $P_{B}=\left\{\left(x_{B_{j}},\omega_{B_{j}}\right)\right\}$. The fused particle set is then obtained as:

(14) $$P^{\prime }=\left\{\left(x_{k},\omega_{k}\right)\right\},\;\omega_{k}=\frac{\omega_{A_{i}}\cdot \omega_{B_{j}}}{\sum_{i,j}\omega_{A_{i}}\omega_{B_{j}}}$$

**Remark** **3.** *This fusion mechanism overcomes the limitations of simple weighted averaging in conventional multi-sensor systems by formulating particle weights as a normalized product of probabilities from collaborating RSUs. This probabilistic integration enhances measurement consistency while maintaining computational efficiency in the particle filtering framework.*

Given that the coordinate systems of the two RSUs have been aligned, the fused particle position $x_{k}$ can be directly computed via a weighted average:

(15) $$x_{k}=\alpha_{A}\sum_{i=1}^{N_{A}}x_{A_{i}}\cdot \omega_{A_{i}}+\alpha_{B}\sum_{j=1}^{N_{B}}x_{B_{i}}\cdot \omega_{B_{i}}$$

Here, $\alpha_{A}$ and $\alpha_{B}$ represent the confidence levels of the two RSUs, both set to 0.5 in this context.

Subsequently, the effective sample size $N_{eff}$ is calculated to determine the necessity of resampling:

(16) $$N_{eff}=\frac{1}{\sum_{i=1}^{N}{\left(\omega_{i}\right)}^{2}}$$

If $N_{eff}<2/N$, resampling is performed, generating a new particle set $P_{n}={\left\{\left(x_{i}^{new},1/N\right)\right\}}_{i=1}^{N}$.

**Remark** **4.** *This resampling criterion introduces an adaptive threshold based on effective sample size, overcoming the limitations of conventional fixed-interval resampling.*

**Figure 5:**
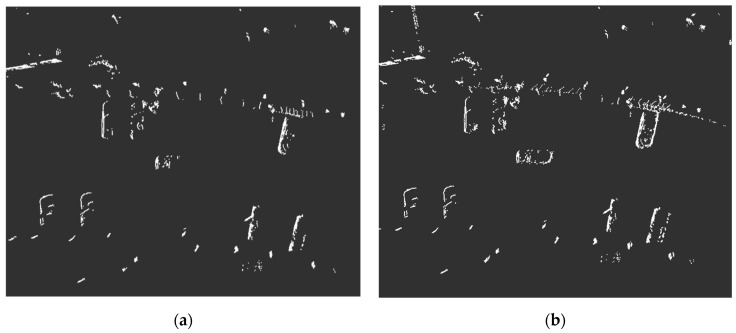
(**a**) Original point cloud. (**b**) Measure the point cloud after fusion.

An experimental evaluation of the measurement fusion process was conducted in a road scenario equipped with two RSUs. The comparative results, illustrated in Figure 5, demonstrate that the fused point clouds exhibit sharper contours and significantly increased density for multiple vehicle targets compared to the unfused local measurements (Figure 5a). This improvement (Figure 5b) provides a richer point cloud for subsequent target state estimation.

#### 2.3.2. State Estimation

To address the issue of discontinuity and lack of ordering in the multi-target state sets generated by Random Finite Set (RFS) filtering, which hinders the formation of continuous target trajectories, this paper introduces a particle labeling strategy applied to the particle set.

The core concept of the particle labeling method is as follows: during each iteration of the particle PHD filter, particles are categorized within the spatial domain, and particles belonging to the same category are assigned an identical label. During the resampling process, offspring particles inherit the label from their parent particles. Following resampling, particles are clustered again. Within each cluster, the predominant label—shared by the majority of particles—is used to associate the cluster with its corresponding cluster from the previous time step. Ultimately, by linking targets that share the same label across iterations, a complete trajectory for each target is constructed. The specific procedural steps of the particle labeling method are described as follows (for *k* ≥ 2):

(1) Prediction

Based on the target motion model, the current particle set is propagated to obtain the predicted particle set, incorporating process noise to enhance particle diversity. The stateparticle is expressed as:

(17) $$x_{k}^{\left(i\right)}=f\left(x_{k-1}^{\left(i\right)}\right)+v_{k-1}^{\left(i\right)}$$

where f(·) denotes the state transition function (e.g., Constant Velocity (CV) or Coordinated Turn (CT) models), and $v_{k-1}^{\left(i\right)}$ represents the process noise.

Given the updated intensity $v_{k-1}\left(x\right)$ at time k−1, the predicted intensity $v_{k|k-1}\left(x\right)$ at time kk is formulated as:

(18) $$v_{k|k-1}\left(x\right)=\sum_{i=1}^{L_{k-1}}\omega_{k-1}^{\left(i\right)}\delta \left(x-x_{k-1}^{\left(i\right)}\right)$$

Furthermore, considering both surviving and newborn targets, the overall predicted intensity becomes:

(19) $$v_{k|k-1}\left(x\right)=\sum_{i=1}^{L_{k-1}}\omega_{P,k|k-1}^{\left(i\right)}\delta \left(x-x_{P,k|k-1}^{\left(i\right)}\right)+\sum_{i=1}^{L_{\gamma ,k}}\omega_{\gamma ,k}^{\left(i\right)}\delta \left(x-x_{\gamma ,k}^{\left(i\right)}\right)$$

with the corresponding components defined by:

(20) $$x_{P,k|k-1}^{\left(i\right)}\sim q_{k}\left(\cdot |x_{k-1}^{\left(i\right)},Z_{k}\right),\;i=1,2,\cdots ,L_{k-1}$$

(21) $$\omega_{P,k|k-1}^{\left(i\right)}=\frac{\omega_{k-1}^{\left(i\right)}P_{S,k}\left(x_{k-1}^{\left(i\right)}\right)f_{k|k-1}\left(x_{P,k|k-1}^{\left(i\right)}|x_{k-1}^{\left(i\right)}\right)}{q_{k}\left(x_{P,k|k-1}^{\left(i\right)}|x_{k-1}^{\left(i\right)},Z_{k}\right)}$$

(22) $$x_{\gamma ,k}^{\left(i\right)}\sim b_{k}\left(\cdot |Z_{k}\right),\;i=1,2,\cdots ,L_{\gamma ,k}$$

(23) $$\omega_{\gamma ,k|k-1}^{\left(i\right)}=\frac{1}{L_{\gamma ,k}}\frac{\gamma_{k}\left(x_{\gamma ,k}^{\left(i\right)}\right)}{b_{k}\left(x_{\gamma ,k}^{\left(i\right)}|Z_{k}\right)}$$

Here, $q_{k}\left(\cdot |x_{k-1}^{\left(i\right)},Z_{k}\right)$ is the importance density for surviving targets, and $b_{k}\left(\cdot |Z_{k}\right)$ is the importance density function for newborn targets.

Concurrently, for i=1, 2, ⋯,M, each particle is assigned a label:

(24) $$L_{k}^{P}\left({\tilde{x}}_{k}^{\left(i\right)}\right)=L_{k}^{NEW}$$

(2) Update

Assuming the predicted intensity at time k is $v_{k|k-1}\left(\cdot \right)$, expressed as:

(25) $$v_{k|k-1}\left(x\right)=\sum_{i=1}^{L_{k|k-1}}\omega_{k|k-1}^{\left(i\right)}\delta \left(x-x_{k|k-1}^{\left(i\right)}\right)$$

then the updated intensity $v_{k}\left(x\right)$ at time kk is given by:

(26) $$v_{k}\left(x\right)=\sum_{i=1}^{L_{k|k-1}}\omega_{k}^{\left(i\right)}\delta \left(x-x_{k}^{\left(i\right)}\right)$$

The weight update formula is:

(27) $$\omega_{k}^{\left(i\right)}=\left[1-p_{D,k}\left(x_{k}^{\left(i\right)}\right)+\sum_{z\in Z_{k}}\frac{\psi_{k,z}\left(x_{k}^{\left(i\right)}\right)}{\kappa_{k}\left(z\right)+\sum_{i=1}^{L_{k|k-1}}\psi_{k,z}\left(x_{k}^{\left(i\right)}\right)\omega_{k|k-1}^{\left(i\right)}}\right]\omega_{k|k-1}^{\left(i\right)}$$

(28) $$\psi_{k,z}\left(x_{k}^{\left(i\right)}\right)=p_{D,k}\left(x_{k}^{\left(i\right)}\right)g_{k}\left(z|x_{k}^{\left(i\right)}\right)$$

(3) Resample

Let $L_{k}=N\times {\hat{N}}_{k}$, where N is the number of particles allocated per target and ${\hat{N}}_{k}$ is the estimated number of targets at the current time. The updated particle set ${\left\{x_{k}^{\left(i\right)},\omega_{k}^{\left(i\right)}\right\}}_{i=1}^{L_{k|k-1}}$ is resampled. During resampling, particles are selected with probabilities proportional to their weights $\omega_{k}^{\left(i\right)}$, yielding a new particle set ${\left\{x_{k}^{\left(i\right)},{\hat{N}}_{k}∕L_{k}\right\}}_{i=1}^{L_{k|k-1}}$, where ${\hat{N}}_{k}∕L_{k}$ is the new weight for each resampled particle.

For the new particles after resampling, labels are inherited from their parent particles. Specifically, if $x_{k}^{\left(j\right)}\in child({\tilde{x}}_{k}^{\left(i\right)})$, then the label is assigned as $L_{k}^{R}\left(x_{k}^{\left(j\right)}\right)=L_{k}^{U}\left({\tilde{x}}_{k-1}^{\left(i\right)}\right)$. This process ensures temporal continuity of labels, thereby maintaining target tracks. The classification after resampling is determined by $\left\{P_{k}^{\left(R,1\right)},P_{k}^{\left(R,2\right)},\cdots ,P_{k}^{\left(R,{\hat{T}}_{k-1}+1\right)}\right\}$.

(4) Target Number and State Estimation

The target number estimate in the SMC-PHD filter is given by:

(29) $${\hat{N}}_{k}=int\left(\sum_{i=1}^{L_{k|k-1}}\omega_{k}^{\left(i\right)}\right)$$

Target states and covariances are determined via k-means clustering applied to the set of weighted particle states and their associated covariances:

(30) $$\left\{\left({\bar{x}}_{k}^{\left(1\right)},S_{k}^{\left(1\right)}\right),\cdots ,\left({\bar{x}}_{k}^{\left({\hat{T}}_{k}\right)},S_{k}^{\left({\hat{T}}_{k}\right)}\right)\right\}$$

During clustering, if two state estimates ${\bar{x}}_{k}^{\left(i\right)}$ and ${\bar{x}}_{k}^{\left(j\right)}$ satisfy the following condition, they are considered too close and potentially belong to the same target, prompting re-clustering based on velocity information:

(31) $$exp\left\{-\frac{1}{2}{\left(H{\bar{x}}_{k}^{\left(i\right)}-H{\bar{x}}_{k}^{\left(j\right)}\right)}^{T}\left(H^{T}S_{k}^{\left(i\right)}H\right)\left(H{\bar{x}}_{k}^{\left(i\right)}-H{\bar{x}}_{k}^{\left(j\right)}\right)\right\}<\gamma$$

where H is the coefficient matrix from the second-order Taylor expansion of the measurement function h(·), and γ is a predefined threshold.

Finally, labels $\left\{L_{k}^{\left(1\right)},L_{k}^{\left(2\right)},\cdots ,L_{k}^{\left({\hat{T}}_{k}\right)}\right\}$ are assigned to the clusters, resulting in the estimated classification $\left\{P_{k}^{\left(1\right)},P_{k}^{\left(2\right)},\cdots ,P_{k}^{\left({\hat{T}}_{k}\right)}\right\}$. For each cluster, the weighted mean is computed as the target state estimate:

(32) $${\hat{x}}_{j}=\frac{\sum_{i\in C_{j}}\omega_{k}^{\left(i\right)}x_{k}^{\left(i\right)}}{\sum_{i\in C_{j}}\omega_{k}^{\left(i\right)}}$$

#### 2.3.3. Trajectory Extraction

At this stage, two cluster sets are obtained:

The combined set from the previous time step’s clusters and the newborn target particle cluster: $\left\{P_{k}^{\left(R,1\right)},P_{k}^{\left(R,2\right)},\cdots ,P_{k}^{\left(R,{\hat{T}}_{k-1}+1\right)}\right\}$. The cluster set at the current time k: $\left\{P_{k}^{\left(1\right)},P_{k}^{\left(2\right)},\cdots ,P_{k}^{\left({\hat{T}}_{k}\right)}\right\}$.

To characterize the particle associations between clusters, two key matrices A and B are defined:

Matrix $A_{g,h}$ captures the particle overlap between current resampled clusters and previous time step clusters:

(33) $$A_{g,h}=\#\left\{i:x_{k}^{\left(i\right)}\in p_{k}^{\left(R,g\right)}\cap p_{k}^{\left(h\right)}\right\}$$

It counts the number of particles in the current resampled cluster $p_{k}^{\left(R,g\right)}$ that also belonged to the previous cluster $p_{k}^{\left(h\right)}$.

Matrix $B_{g,h}$ reflects the distribution of offspring particles after resampling:

(34) $$B_{g,h}=\#\left\{i:child\left(x_{k}^{\left(i\right)}\right)\in p_{k}^{\left(R,g\right)}\cap p_{k}^{\left(h\right)}\right\}$$

It counts the number of particles in $p_{k}^{\left(R,g\right)}$ whose parent particles came from the previous cluster $p_{k}^{\left(h\right)}$.

Using these matrices, trajectory extraction is performed as follows:

(1) Surviving Target Identification

Ideal Threshold: Under accurate clustering, the number of particles corresponding to each target should satisfy:

(35) $$\sum_{g=1}^{{\hat{T}}_{k-1}}A_{g,h}\approx N$$

If $A_{g,h}\approx N$, it indicates that target g from the previous time step likely remains alive.

Threshold-based Judgment: A threshold $\epsilon_{1}$ is set. If for a previous target g,

(36) $$\sum_{h=1}^{{\hat{T}}_{k}}A_{g,h}=\epsilon_{1}N$$

then target h is considered to have disappeared.

(2) Newborn Target Identification

A threshold $\epsilon_{2}$ is defined. If the number of newborn target particles within any current cluster exceeds $\epsilon_{2}N$, a new target is declared.

(3) Spawned Target Handling

If the particles from a previous target g split into multiple clusters after resampling (e.g., due to target spawning), the corresponding elements in matrix A might exhibit similar particle counts for these clusters. Matrix B is utilized for further discrimination: offspring particles of a surviving target should predominantly originate from itself (indicated by a larger $B_{g,h}$), whereas offspring particles associated with a spawned target are likely to be fewer.

In summary, by applying Equations (33), (35) and (36), the target state estimates and their associated trajectories are obtained.

Following the detailed exposition of the core principles and sequential phases—Prediction, Update, Resample, and Trajectory Extraction—the particle-labeled SMC-PHD tracking algorithm is concisely summarized in the Algorithm 1. This formulation encapsulates the key procedures and data flow, providing a clear blueprint for implementation.
**Algorithm 1** Particle-Labeled PHD Filter1: **procedure** MAIN (InitialParticleSet, MeasurementSequence) 2:  **for** k = 2 **to** K **do** 3:    **PREDICTION**(k**)** 4:    **UPDATE**(k**)** 5:    **RESAMPLE**(k**)** 6:    **ESTIMATE_TARGETS**(k**)** 7:    **EXTRACT_TRAJECTORIES**(k**)** 8:  **end for** 9: **end procedure** 10: **procedure PREDICTION**(k**)** 11:  **for** $i\;=\;1\;\mathrm{to}\;L_{k-1}$** do** 12:    $x_{k}^{\left(i\right)}=f\left(x_{k-1}^{\left(i\right)}\right)+v_{k-1}^{\left(i\right)}$ 13:    $\omega_{P,k|k-1}^{\left(i\right)}$$\;=\;(\omega_{k-1}^{\left(i\right)}$ × $P_{S,k}$ × $f_{k|k-1}$$)/q_{k}$ 14:    $x_{P,k|k-1}^{\left(i\right)}$$\;∼\;q_{k}\left(\cdot |x_{k-1}^{\left(i\right)},Z_{k}\right)$ 15:  **end for** 16:  $\mathrm{for}\;i\;=\;1\;\mathrm{to}\;L_{\gamma ,k}$** do** 17:    $x_{\gamma ,k}^{\left(i\right)}$$\;∼\;b_{k}\left(\cdot |Z_{k}\right)$ 18:    $\omega_{\gamma ,k|k-1}^{\left(i\right)}$$\;=\;(1/\gamma_{k}$$)\;\times \;\gamma_{k}/b_{k}$ 19:    $L_{k}^{P}\left({\tilde{x}}_{k}^{\left(i\right)}\right)$$\;=\;L_{k}^{NEW}$ 20:  **end for** 21:  **Combine predicted particle sets** 22: **end procedure** 23: **procedure UPDATE**(k**)** 24:  $\mathrm{for}\;i\;=\;1\;\mathrm{to}\;L_{k|k-1}$** do** 25:    $\mathrm{Calculate}\;\psi_{k,z}\left(x_{k}^{\left(i\right)}\right)$$\;=\;p_{D,k}$ × $g_{k}$ 26:    $\mathrm{Update}\;\mathrm{weight}\;\omega_{k}^{\left(i\right)}$$\;=\;[1-p_{D,k}$$\;+\;\sum_{z\in Z_{k}}\psi_{k,z}/(\kappa_{k}+\sum_{i=1}^{L_{k|k-1}}\psi_{k,z}\times \omega_{k|k-1}^{\left(i\right)})]$ 27:    $\psi_{k,z}\left(x_{k}^{\left(i\right)}\right)$$\;=\;p_{D,k}\times g_{k}$ 28:  **end for** 29: **end procedure** 30: **procedure RESAMPLE**(k**)** 31:  $L_{k}$ = N ×$\;\mathrm{round}(\sum_{i=1}^{L_{k|k-1}}\omega_{k}^{\left(i\right)})$ 32:  $\mathrm{Resample}\;L_{k}$$\;\mathrm{particles}\;\mathrm{with}\;\mathrm{probability}\;\propto \;\omega_{k}^{\left(i\right)}$ 33:  $\mathrm{Assign}\;\mathrm{new}\;\mathrm{weights}\;\omega_{k}^{\left(i\right)}$$\;=\;{\hat{N}}_{k}/L_{k}$ 34:  $\mathrm{for}\;\mathrm{each}\;\mathrm{resampled}\;\mathrm{particle}\;x_{k}^{\left(j\right)}$** do** 35:    $\mathrm{Inherit}\;\mathrm{label}:\;L_{k}^{R}\left(x_{k}^{\left(j\right)}\right)$$\;=\;L_{k}^{U}$ (parent particle**)** 36:  **end for** 37:  $\mathrm{Cluster}\;\mathrm{resampled}\;\mathrm{particles}\;\mathrm{into}\;P_{k}^{\left(R,g\right)}$ 38: **end procedure** 39: **procedure ESTIMATE_TARGETS**(k**)** 40:  **Perform k-means clustering on weighted particles** 41:  $\mathrm{for}\;\mathrm{each}\;\mathrm{cluster}\;C_{j}$** do** 42:    **if cluster states too close then re-cluster by velocity** 43:    $\mathrm{Compute}\;\mathrm{estimated}\;\mathrm{state}\;{\hat{x}}_{j}$$\;=\;\sum_{i\in C_{j}}\omega x/\sum_{i\in C_{j}}\omega$ 44:    $\mathrm{Assign}\;\mathrm{label}\;L_{k}^{\left(j\right)}$$\;\mathrm{to}\;\mathrm{cluster}\;C_{j}$ 45:  **end for** 46:  **Output clusters** [10] 47: **end procedure** 48: **procedure EXTRACT_TRAJECTORIES**(k**)** 49:  **Compute matrices** A **and** B **using Equations** (33) **and** (34) 50:  **for each previous cluster** g **do** 51:    $\mathrm{if}\;\sum_{g=1}^{{\hat{T}}_{k-1}}A_{g,h}\approx N$** then target survives** 52:    $\mathrm{if}\;\sum_{h=1}^{{\hat{T}}_{k}}A_{g,h}=\epsilon_{1}N$** then target disappears** 53:  **end for** 54:  **for each current cluster h do** 55:    $\mathrm{if}\;\mathrm{newborn}\;\mathrm{particles}\;>\;\epsilon_{2}N$** then new target declared** 56:  **end for** 57:  **Use** matrix B **to handle target spawning cases** 58:  **Link targets with same labels across time steps** 59: **end procedure**

## 3. Results

This section further developed a roadside collaborative multi-target tracking system suitable for Foggy conditions, constructed an intelligent roadside hardware platform, and verified the effectiveness of the system in noise suppression and tracking continuity.

### 3.1. Experimental Platfrom

To validate the denoising efficacy and tracking performance of the proposed intelligent roadside multi-target tracking system under real-world Foggy conditions, field experiments were conducted in actual traffic scenarios using the Intelligent roadside platform (depicted in Figure 7). Each RSU integrated Lidar, Camera, computational module, GNSS/RTK and communication units. Each LiDAR timestamp served as the benchmark with a 10 Hz sampling rate. Based on the PTP method, the time information of the satellite atomic clock is received through GPS and transmitted to the time synchronization box to complete the timing work of the PTP master clock and achieve time synchronization of various sensors. The computational module leveraged the NVIDIA Jetson AGX Orin Developer Kit as the core processing unit, which incorporates a high-performance, power-efficient processor capable of real-time execution of computationally intensive algorithms. Regarding software configuration, the platform runs on Ubuntu 20.04 and utilizes the Robot Operating System (ROS1) as the software development framework for programming and system integration.

**Figure 6:**
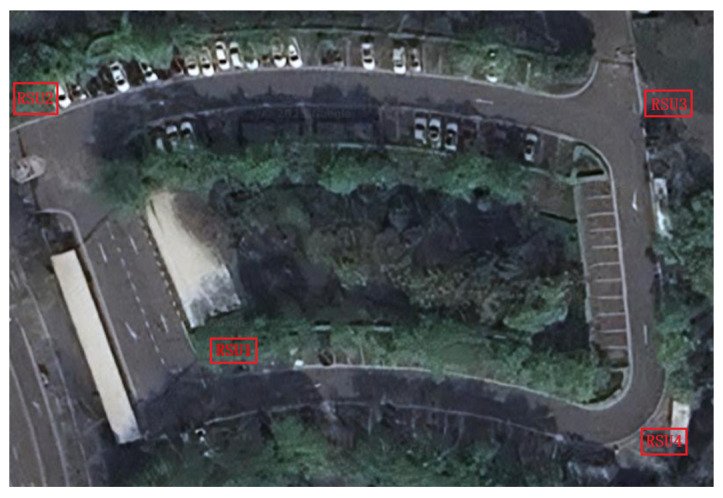
Experimental site BEV.

**Figure 7:**
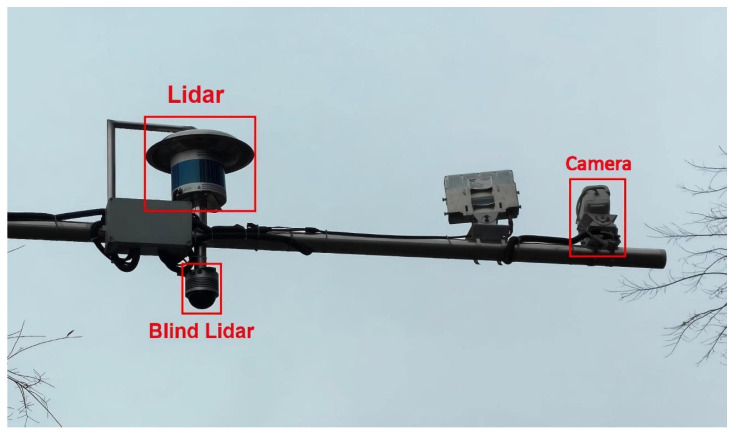
Intelligent Roadside Experimental Platform.

The experimental testbed is illustrated in Figure 6, comprising four RSUs deployed at the four corners of the road segment. The baseline separation between RSU1 and RSU2 is 90 m, while the distance between RSU1 and RSU4 is 150 m. The intelligent roadside perception platform is depicted in Figure 7, integrating an 80-channel LiDAR, a 32-channel blind-spot LiDAR, and IP cameras. All sensors are mounted at a uniform height of 6 m above the roadway centerline. The blind-spot and primary roadside LiDARs are vertically collocated to enable streamlined parameter calibration and point cloud fusion. Furthermore, the sensors are configured within a unified IP subnet and interfaced with edge computing units via Gigabit Ethernet for real-time data processing. Inter-RSU coordination is achieved through V2X communication. This experimental configuration was employed to validate the feasibility of the proposed tracking framework.

#### 3.1.1. Point Cloud Denoising

**Figure 8:**
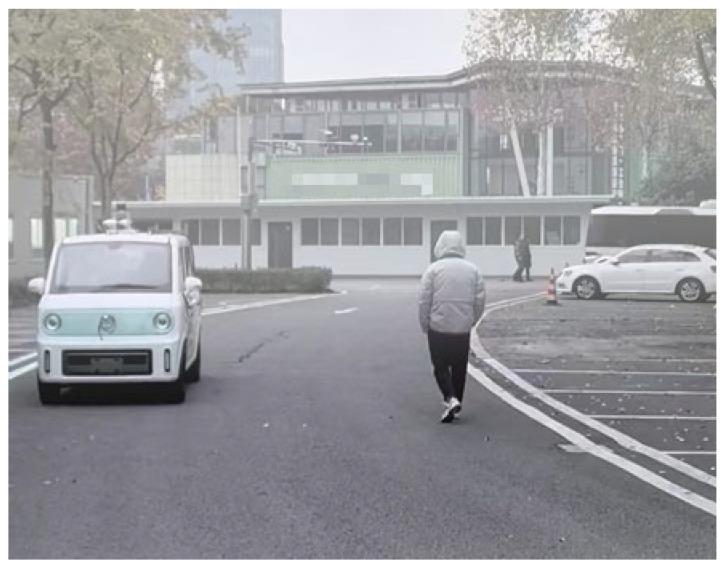
Experimental scenario.

To validate the effectiveness of the proposed noise reduction method, point cloud data collected under three distinct fog concentrations during roadside platform experiments were employed. Following meteorological standards [36], light fog (visibility: 1000–10,000 m), moderate fog (500–1000 m), and heavy fog (<500 m) were systematically evaluated. The meteorological parameters acquired on the day of the experiment are presented in Table 2. The experimental scene was shown in Figure 8.

**Figure 9:**
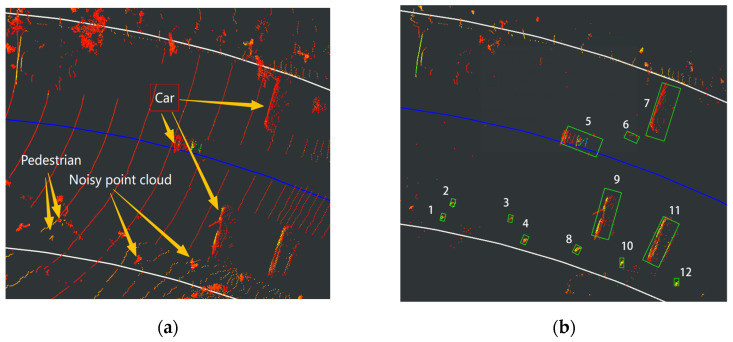

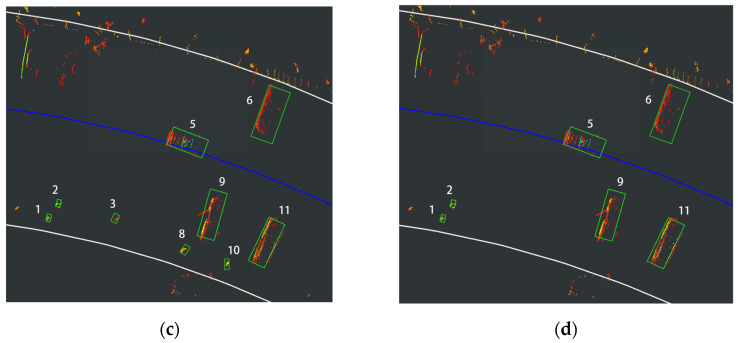
Results of Experiment: (**a**) original point cloud; (**b**) unprocessed clustering results. (**c**) Statistical filtering results. (**d**) Proposed method for filtering results.

A comparative evaluation was conducted between the proposed method and statistical filtering for point cloud denoising using 120 frames of data. As shown in Figure 9, the raw fog-affected point cloud (a) contains significant noise-induced artifacts. While basic preprocessing (b) fails to eliminate false targets (with only ID 1, 2, 5, 7, 9, 11 being valid objects), statistical filtering (c) shows limited improvement. The proposed method (d) demonstrates superior denoising performance, effectively suppressing false targets while preserving true detections.

It is observed from Figure 9b that IDs 1, 2, 5, 7, 9 and 11 are valid targets. The proposed denoising method successfully eliminates all false detections, whereas the statistical filtering approach, despite removing some false targets, fails to eliminate others (e.g., Targets 3, 8, and 10). This demonstrates a clear improvement in denoising effectiveness achieved by the proposed method.

For quantitative evaluation of the denoising performance, the total number of targets is defined as all objects (including both valid and false detections) present in the road area per frame. Let $N_{e}$ represent the number of false detections and N the total number of targets. The target detection accuracy n is defined as:

(37) $$n=\frac{N-N_{e}}{N}\times 100\%$$

We selected 120 frames of images, and the final statistical results are shown Table 3 and Table 4.

Compared with the statistical filtering method, the target detection accuracy after denoising by the method proposed in this paper has increased by 8%.

Subsequently, experiments were conducted using the same method under heavy fog conditions, with the corresponding experimental results presented as follows.

It is observed from the experimental results in Table 4 that the detection accuracy rate of the proposed method is 29% higher than that of statistical filtering under heavy fog conditions. This can be explained by the fact that the statistical filtering denoising method filters out outliers by analyzing the distance distribution characteristics in the neighborhood of point clouds and dynamically setting the standard deviation threshold. However, as the fog concentration increases, valid points in low-density point cloud areas tend to be over-filtered. In contrast, the proposed method benefits from the introduction of intensity features by the bilateral filter, enabling better preservation of details in low-density point cloud areas and distinguishing fog noise point clouds in high-noise areas.

#### 3.1.2. Multi-Target Tracking Experiments and Analysis

To evaluate the performance of the proposed 3D multi-object tracking algorithm, we conduct systematic experimental validations on the dataset, which is collected by the above vehicle road collaboration platform. The dataset was decomposed into straight and curved sections, and experiments were conducted based on different road conditions, with experimental speeds ranging from 10 km/h to 80 km/h, to verify the effectiveness of the proposed tracking method.

A rigorously validated multi-dimensional evaluation framework is employed to holistically quantify the precision and robustness of tracking algorithms. Central to this methodology is the Higher-order Tracking Accuracy (HOTA) [37], which delivers a balanced performance characterization under complex operational conditions by jointly optimizing detection, association, and localization fidelity. As a supplement to the core indicators, auxiliary verification is carried out through several other key indicators: Multi target Tracking Accuracy (MOTA), Multi target Tracking Precision (MOTP), Identity Consistency (IDF1), Trajectory Integrity (MT), Trajectory Loss (ML), and Identity Switching Rate (IDs). This dual index method ensures comprehensive evaluation while reducing the scenario specific bias inherent in single index evaluation.

Given that existing research typically focuses on optimizing specific road scenarios, we conducted a detailed comparison between our method and the current state-of-the-art and most classic solutions for both straight and curved categories. The results indicate that this method outperforms existing technologies in multiple key indicators. The detailed evaluation results are shown in Table 5 and Table 6. The up arrow (↑) indicates that the higher the value, the better, and the down arrow (↓) indicates that the lower the value, the better.

- Straight Scenario

**Figure 10:**
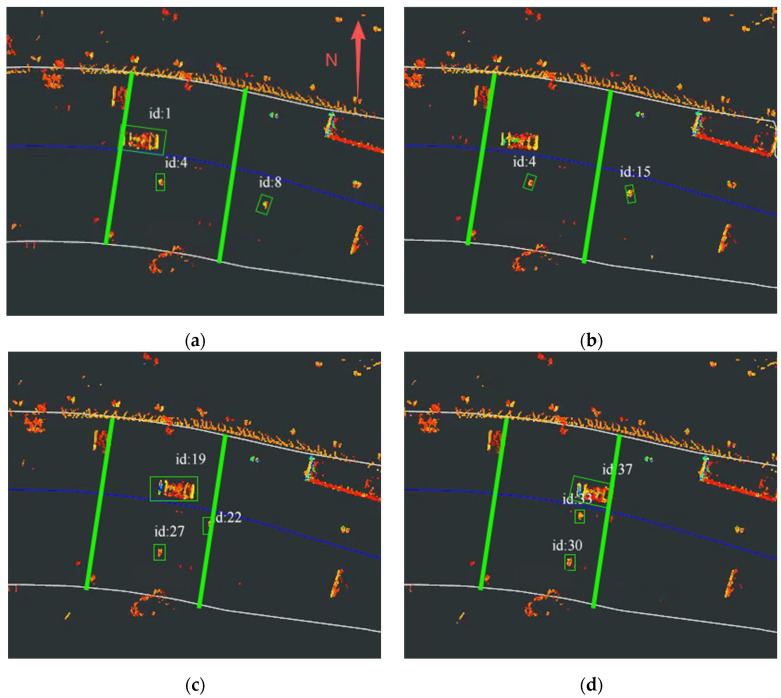
Tracking result of JPDA: (**a**) Target tracking status at 8:52:49. (**b**) Target tracking status at 8:52:50. (**c**) Target tracking status at 8:52:51. (**d**) Target tracking status at 8:52:52.

**Figure 11:**
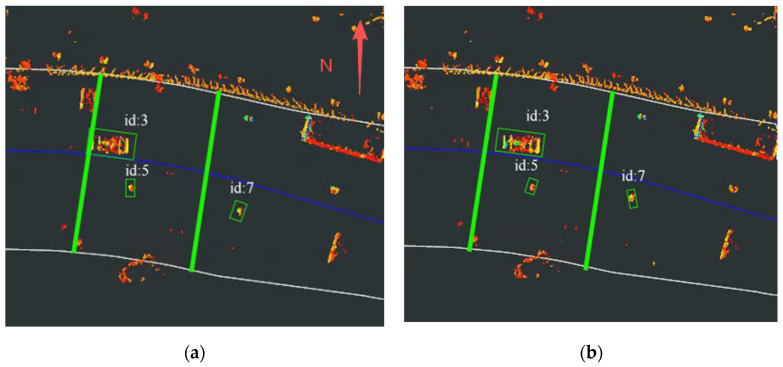

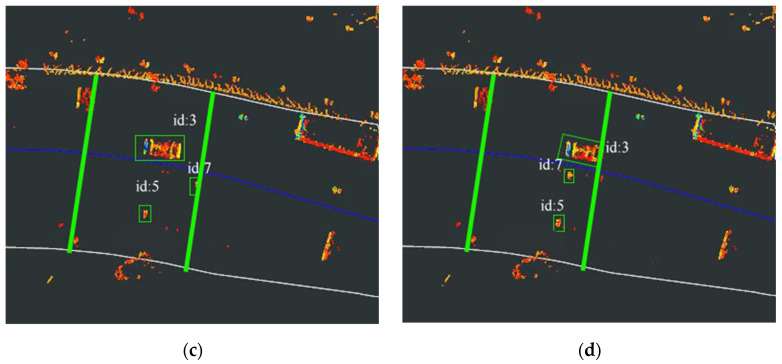
Tracking result of proposed method: (**a**) Target tracking status at 8:52:49. (**b**) Target tracking status at 8:52:50. (**c**) Target tracking status at 8:52:51. (**d**) Target tracking status at 8:52:52.

We selected one of the experimental segments for display. The experimental results at different time instants of the JPDA-based method and the proposed method are illustrated in Figure 10 and Figure 11, respectively. Point clouds were fused from detections by RSU2 and RSU3, each with a LiDAR perception range of (0, 50 m). The vehicle traveled east to west at 10 km/h. The yellow line in Figures indicate the transition zone between RSU2 and RSU3, spanning approximately 20 m. The green box represents the target tracking box.

As shown in Figure 10a–d, the ID of the experimental vehicle changes or disappears intermittently, and the pedestrian target ID also fluctuates. This indicates that targets are not consistently tracked within the transition zone, demonstrating the poor stability of the JPDA-based tracking method during cross-domain tracking in straight-road scenarios.

In contrast, Figure 11a–d show that both vehicle and pedestrian targets maintain consistent IDs within the transition zone, confirming the proposed method’s capability for continuous cross-domain tracking. This demonstrates the superior performance of the proposed method over JPDA in straight-road scenarios.

As shown in the comprehensive comparison in Table 5, our method achieved highly competitive results on the test set. This method demonstrates significant advantages in multiple key indicators, particularly in HOTA, MOTA, IDF1, and IDs indicators. This outstanding performance is mainly attributed to the synergistic effect of multiple RSUs. Multi-object state estimation based on particle probability assumption density filtering framework, using particle identification to associate target states and accurately distinguish occluded objects from exiting objects. Ultimately, while reducing the IDSW index, the HOTA index was significantly improved.

- Curved Scenario

**Figure 12:**
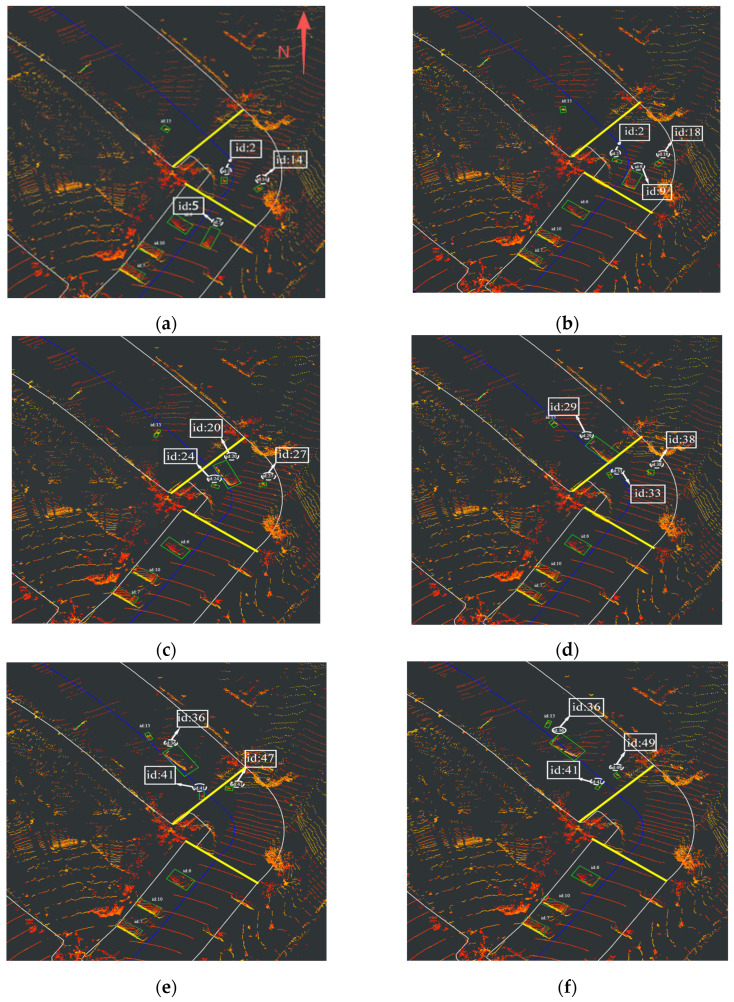
Tracking result of data association: (**a**) Target tracking status at 7:19:12. (**b**) Target tracking status at 7:19:13. (**c**) Target tracking status at 7:19:14. (**d**) Target tracking status at 7:19:15. (**e**) Target tracking status at 7:19:16. (**f**) Target tracking status at 7:19:17.

Similarly, a representative sequence is selected to demonstrate the superior multi-object tracking performance of the proposed method, Figure 12 depicts the inter-RSU handoff tracking performance of the JPDA method. In this curved-road scenario, the test vehicle traversed the bend at a constant velocity of 20 km/h, while the pedestrian exited the curvature at a walking speed of 4 km/h. The solid yellow line denotes the curved segment, which corresponds to the transition zone.

Similarly, in Figure 12a–f, both the vehicle and pedestrian undergo ID switches within the transition zone, indicating the JPDA method’s failure to maintain consistent tracking through the curved section.

**Figure 13:**
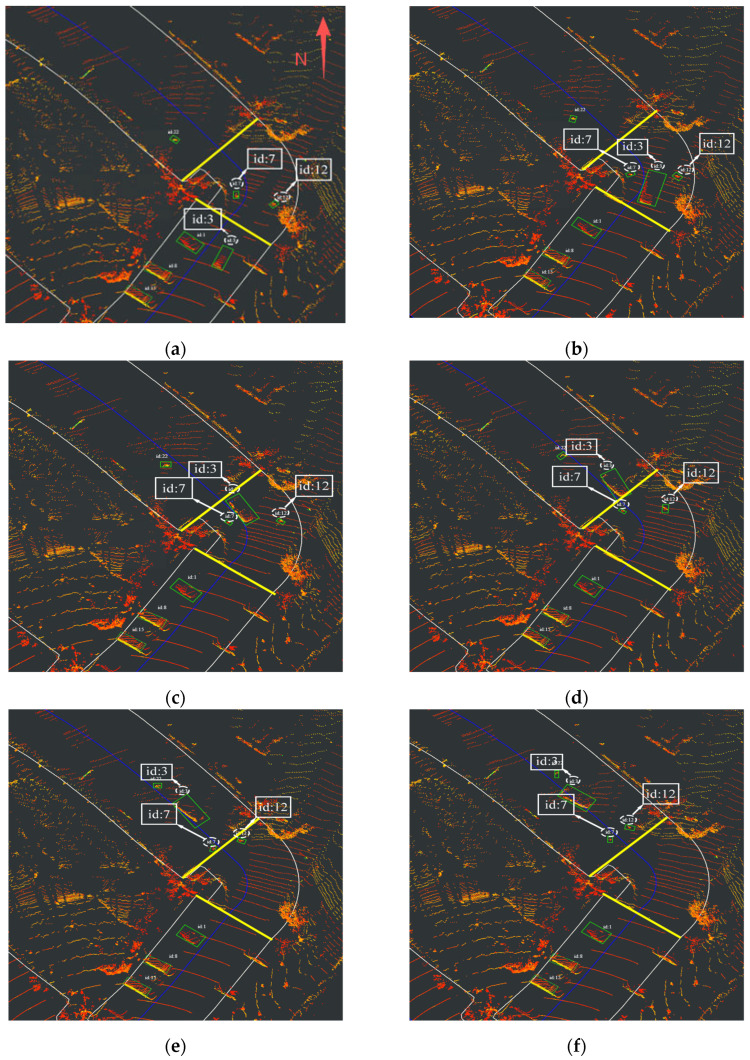
Tracking result of proposed method (**a**) Target tracking status at 7:19:12. (**b**) Target tracking status at 7:19:13. (**c**) Target tracking status at 7:19:14. (**d**) Target tracking status at 7:19:15. (**e**) Target tracking status at 7:19:16. (**f**) Target tracking status at 7:19:17.

In Figure 13a, ID 3 corresponds to passenger vehicles, and ID 7 and ID 12 represent two pedestrians, respectively.

In contrast, Figure 12a–f demonstrate the operational mechanism of the proposed method: RSU4 continuously broadcasts the vehicle’s measurement data to neighboring units. When the target enters the detection range of RSU2, the received measurements are correlated with local observations. Upon successful association, the target state estimate is updated using the new measurements, achieving seamless cross-RSU cooperative tracking. The results confirm that the proposed tracking method maintains stable and continuous tracking throughout the entire curve negotiation process.

In Figure 13a–f, both vehicle and pedestrian targets maintain consistent IDs within the transition zone, demonstrating continuous tracking capability throughout the curved section. These results validate the superior performance of the proposed multi-target tracking method over JPDA in curved road scenarios.

The category of curves has characteristics such as complex motion models, frequent occlusions, and limited sensor perspectives, which make it a major challenge to distinguish occluded targets when exiting objects. As shown in Table 6, for the curve tracking tasks, the proposed method achieves first place in HOTA, MOTA, MOTP, MT, and IDs metrics, and secures second place in IDF1 and ML metrics. These results strongly demonstrate the effectiveness of this method in tracking nonlinear, small-scale, and easily occluded targets in curved scenes.

### 3.2. Computational Performance and Analysis

**Figure 14:**
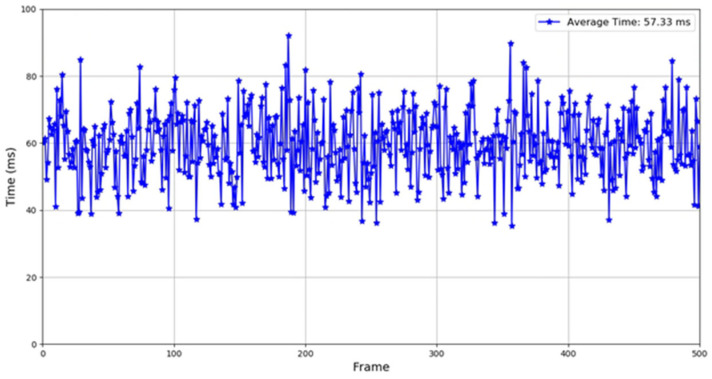
System runtime.

To verify the timeliness of the multi-target tracking method proposed in this article, we conducted experiments in actual scenarios. Specifically, we deployed the intelligent roadside experimental platform in a real traffic environment, ran the RSU collaborative multi-target tracking algorithm proposed in this paper, performed 500 cross domain multi-target tracking tasks, and accurately recorded the average processing time of each tracking task. The experimental results are shown in Figure 14. Considering that the operating frequency of the laser radar used is 10 Hz and its sampling period is 100 ms, the average operating time of the system proposed in this paper is only 57.33 ms, which is significantly shorter than the sampling period of the laser radar. This fully demonstrates that the method proposed in this article can meet the strict real-time requirements of intelligent roadside systems for multi-target tracking while ensuring processing accuracy, ensuring real-time perception of environmental changes by roadside units and providing a reliable basis for subsequent decision-making and control.

## 4. Discussion

While the proposed adaptive point cloud denoising and multi-RSU cooperative tracking framework has demonstrated substantial improvements in noise suppression, trajectory continuity, and inter-RSU label consistency under Foggy conditions, several limitations remain.

First, the dynamic parameter tuning within the multi-constraint filtering model relies on local noise statistics, which exhibits limited capability in capturing the inherent non-uniform noise characteristics of fog, particularly within transition zones where fog density varies. Consequently, the real-time efficacy and accuracy of Gaussian kernel scaling may be constrained by simplifying assumptions, potentially resulting in residual noise or over-smoothing of target details. Future work could investigate a self-supervised learning framework grounded in explicit fog noise modeling, leveraging deep neural networks to autonomously extract local topological features and implicit noise distribution patterns directly from fog-corrupted point clouds.

Second, although the identifier mechanism based on measurement fusion and particle filtering enhances the robustness of cross-RSU target association, challenges persist under extreme conditions, such as severely degraded visibility or extended occlusions, where observation gaps may still induce trajectory fragmentation. Furthermore, the computational overhead and latency of the current approach in dense-traffic scenarios require optimization for scalable deployment. A promising avenue involves modeling the spatio-temporal topology across RSUs using Graph Neural Networks (GNNs), thereby exploiting perceptual context to reinforce target identity reasoning within the sensor network. Additionally, future efforts should focus on developing lightweight particle filter variants or harnessing edge computing acceleration techniques to meet the stringent real-time constraints of large-scale intelligent transportation system deployments.

## 5. Conclusions

As a critical component of ITS, RSUs can provide continuous and wide-area environmental information for vehicles through multi-node collaborative perception. However, their perceptual performance in adverse weather conditions such as fog is often compromised by low visibility and sensor noise interference, leading to degraded tracking accuracy and ineffective cross-domain data fusion. To address challenges including limited sensing range, low tracking efficiency, and insufficient robustness in multi-target tracking under Foggy conditions, this paper proposes a method integrating adaptive point cloud denoising and multi-RSU collaboration. First, localized noise modeling is employed to dynamically adjust filtering parameters, and a multi-constraint filtering model is constructed by integrating point cloud spatial distribution, intensity gradient, and edge features. This enables rain-fog noise to be effectively suppressed while target details are preserved, thereby reducing the false detection rate. Second, to overcome limitations such as low tracking accuracy and a restricted perception range in roadside sensing systems under foggy conditions, cross-domain fusion of heterogeneous measurements is utilized for long-range perception. This is followed by the incorporation of an identifier inheritance mechanism from particle filtering and cross-unit state transfer, ensuring continuous target trajectory tracking and significantly enhancing cross-domain tracking robustness in complex scenarios.

A multi-target tracking system for intelligent RSUs in Foggy scenarios was designed and implemented. Extensive experiments were conducted using an intelligent roadside platform in real-world fog-affected traffic environments to validate the accuracy and real-time performance of the proposed algorithm. Experimental results demonstrate that the proposed method improves the target detection accuracy by 8% and 29%, respectively compared to statistical filtering methods after removing fog noise under thin and thick fog conditions. At the same time, this method performs well in tracking multi-class targets, surpassing existing state-of-the-art methods, especially in high-order evaluation indicators such as HOTA, MOTA, and IDs.

Nevertheless, the proposed adaptive point cloud denoising and multi-RSU cooperative tracking framework still exhibits certain limitations. For instance, dynamic parameter tuning based on local noise statistics may inadequately capture fog’s inherent non-uniformity—particularly in density transition zones—potentially constraining Gaussian kernel scaling accuracy and causing residual noise or target detail over-smoothing. Additionally, while the measurement fusion and particle filtering identifier enhances cross-RSU association robustness, trajectory fragmentation may persist under extreme visibility degradation or prolonged occlusion. To maintain lightweight design and align with practical deployment constraints, we do not currently implement advanced noise modeling or optimize computational overhead for dense traffic. Future work will focus on enhancing low-quality data processing and association resilience: specifically, developing self-supervised fog noise modeling to refine point cloud denoising, and leveraging graph neural networks to exploit spatio-temporal topology for robust identity reasoning under complex environmental conditions.

## Figures and Tables

**Table 1:** RS-RubyLite Performance Parameter.

Parameter	Specifications
Lines	80
Range	230 m (160 m@10% NIST)
Range Precision (Typical)	Up to ±3 cm
Frame Rate	10 Hz/20 Hz
Horizontal FOV	360°
Vertical FOV	40° (−25°~+15°)
Horizontal Resolution	[Balance] 0.2°/0.4° [High performance] 0.1°/0.2°
Vertical Resolution	Up to 0.1°

**Table 2:** Meteorological Parameters.

Parameter	Value
Horizontal Visibility/m	500~3000
Temperature Range/°C	6~13
Relative Humidity/%	65~76
Wind Speed/km/h	8–14

**Table 3:** Denoising results of actual scenes under mist.

Noise Reduction Methods	Total Target Number	Number of False Positives	Accuracy Rate (%)
Unprocessed	757	210	72%
Statistical filtering	671	124	82%
Proposed method	564	57	90%

**Table 4:** Denoising Results of Actual Scenes in Thick Fog.

Noise Reduction Methods	Total Target Number	Number of False Positives	Accuracy Rate (%)
Unprocessed	384	313	18%
Statistical filtering	415	249	40%
Proposed method	336	104	69%

**Table 5:** Evaluation result of Straight Scenario.

Methods	HOTA ↑	MOTA ↑	MOTP ↑	IDF1 ↑	MT ↑	ML ↓	IDs ↓
BcMODT [38]	72.64	83.18	85.82	62.96	44.69	12.58	89
C-TwiX [39]	78.91	88.56	85.46	56.82	49.32	14.92	241
JPDA	69.88	75.23	72.14	51.19	41.51	30.05	23
Ours	82.76	89.69	87.30	66.57	46.74	11.63	52

**↑** indicate that the higher the numerical value, the better the performance. Conversely, **↓** indicate that the lower the numerical value, the better the performance.

**Table 6:** Evaluation result of Curved Scenario.

Methods	HOTA ↑	MOTA ↑	MOTP ↑	IDF1 ↑	MT ↑	ML ↓	IDs ↓
BcMODT [38]	54.07	55.13	75.87	65.46	35.98	22.65	167
C-TwiX [39]	58.94	57.95	73.61	72.63	33.74	21.07	239
JPDA	42.73	48.72	65.42	62.88	31.85	29.37	380
Ours	61.42	62.73	78.47	70.71	38.46	22.41	129

## Data Availability

The data presented in this study are available on request from the corresponding author due to legal and privacy restrictions.
